# Influence of hypercholesterolemia and diabetes on long-term outcome in patients with stable coronary artery disease receiving percutaneous coronary intervention

**DOI:** 10.1097/MD.0000000000016927

**Published:** 2019-08-23

**Authors:** Mao-Jen Lin, Yu-Jun Chang, Chun-Yu Chen, Chia-Chen Huang, Tzu-Yao Chuang, Han-Ping Wu

**Affiliations:** a Division of Cardiology, Department of Medicine, Taichung Tzu Chi Hospital, The Buddhist Tzu Chi Medical Foundation, Taichung; b Department of Medicine, School of Medicine, Tzu Chi University, Hualien; c Laboratory of Epidemiology and Biostatistics, Changhua Christian Hospital, Changhua; d Department of Pediatric Emergency Medicine, China Medical University Children's Hospital; e Department of Medicine, College of Medicine, China Medical University; f Department of Public Health, Chung Shan Medical University; g Department of Pediatrics, Children's Hospital, China Medical University; h Department of Medical Research, China Medical University Children's Hospital, China Medical University, Taichung, Taiwan.

**Keywords:** diabetes mellitus, hypercholesterolemia, percutaneous coronary intervention

## Abstract

Coronary artery disease (CAD) is a life-threatening medical emergency which needs urgent medical attention. Percutaneous coronary intervention (PCI) is common and necessary for patients with CAD. The effect of hypercholesterolemia and diabetes on long-term outcomes in patients with stable CAD receiving PCI is unclear.

In this study, patients with stable CAD who underwent PCI were prospectively divided into 4 groups according to the presence or absence of diabetes or hypercholesterolemia. Clinical characteristics, risk factors, medications, angiographic findings, and outcome predictors were analyzed and long-term outcomes compared between groups.

Of the 1676 patients studied, those with hypercholesterolemia and diabetes had the highest all-cause mortality rate after PCI (*P* < .01); those with diabetes only had the highest cardiovascular (CV) mortality (*P* < .01). However, the 4 groups did not differ in rates of myocardial infarction (MI) or repeated PCI. In Kaplan–Meier survival analysis, patients with diabetes only had the highest rates of all-cause mortality and CV mortality (both *P* < .001). In the Cox proportional hazard model, patients with both hypercholesterolemia and diabetes had the highest risk of all-cause mortality (hazard ratio: 1.70), but groups did not differ in rates of MI, CV mortality, and repeated PCI.

With or without hypercholesterolemia, diabetes adversely impacts long-term outcomes in patients receiving PCI. Diabetes mellitus seemed to be a more hazardous outcome predictor than hypercholesterolemia. Hypercholesterolemia and diabetes seemed to have an additive effect on all-cause mortality in patients after receiving PCI.

## 1 Introduction

Percutaneous coronary intervention (PCI) is an important therapeutic strategy in patients with coronary artery disease (CAD). Diabetes mellitus (DM) and hypercholesterolemia are major risk factors for coronary atherosclerosis, and also affect outcomes in CAD patients undergoing PCI.^[1–5]^ The impact of DM on outcomes has been well studied in patients with acute coronary syndrome (ACS) undergoing PCI. After undergoing PCI, diabetic patients with ACS had worse short-term and mid-term outcomes than nondiabetic patients with ACS^[6–9]^. On the other hand, elevated low-density lipoprotein (LDL) level on admission is associated with increased in-hospital mortality in diabetic, but not nondiabetic, patients treated with PCI for ST-segment (the segment connects the QRS complex and T wave in electrocardiography) elevation myocardial infarction.^[10]^ However, the isolated and combined effect of hypercholesterolemia and DM on long-term prognosis in stable CAD patients undergoing PCI is still obscure. The purpose of this study is to clarify and to compare long-term outcomes among 4 groups of patients: patients with both DM and dyslipidemia, with DM only, with dyslipidemia only, and without dyslipidemia or DM. In addition, we also aimed to perform an advanced analysis to identify the adverse predictors of clinical outcomes among these 4 groups.

## 2 Methods

### 2.1 Study population

This was a prospective cohort study from 2007 to 2015. We recruited consecutive patients with stable CAD aged 20 to 85 years from the inpatient clinic of Taichung Tzu Chi Hospital, Taiwan. All patients were divided into 4 groups: patients without DM or hypercholesterolemia (control group), patients with DM alone, patients with hypercholesterolemia alone, and patients with both hypercholesterolemia and DM. Patients with scheduled PCI, end-stage heart failure, and malignancy were excluded from this study. Most patients received regular follow-up through the outpatient department. For the few patients lost to follow-up, a telephone call was usually used to contact the patients or their families. For each patient, a survey of cardiovascular (CV) mortality, all-cause mortality, myocardial infarction (MI), and repeated PCI procedures was conducted at the end of the study. The study was approved by the Institutional Review Board of Taichung Tzu Chi Hospital. All methods were performed in accordance with the relevant guidelines and regulations. The data were collected, reviewed, de-identified, and anonymously analyzed by the authors, and written informed consent was obtained from all participants.

### 2.2 Data synthesis, definition, and analysis

Data collected included general clinical characteristics, including body habitus; biochemical profiles; coronary angiographic findings from cardiac catheterization; exposed risk factors; therapeutic strategies such as drug medications prescribed after PCI; and invasive procedures, including balloon angioplasty, bare metal stent deployment, or drug-eluting stent (DES) deployment. Diabetes was defined as a fasting plasma glucose level of >126 mg/dL, or a casual plasma glucose level of >200 mg/dL, or a hemoglobin A1c level of >6.5%.^[11]^ Hypercholesterolemia was defined as a serum cholesterol level of >200 mg/dL or an LDL level of >100 mg/dL.^[12,13]^ Hypertension (HT) was defined as a usual blood pressure (BP) of 140/90 mm Hg or higher, BP levels for which the benefits of pharmacologic treatment have been definitely established.^[14]^ Chronic kidney disease (CKD) was defined as an estimated glomerular filtration rate of <60 mL/min/1.73 m^2^, which was equal to or greater than CKD stage 3.^[15]^ Left ventricular function was evaluated through angiographic ventriculography or nuclear ventriculography. For the angiographic and hemodynamic data, the central aortic pressure was obtained by using a pigtail catheter during a coronary angiography. Coronary angiographic findings were analyzed, including number of diseased vessels, number of lesions, and lesion locations; lesion location, severity, and complexity were evaluated via the Synergy between PCI with Taxus and cardiac surgery score (SYNTAX score).^[16]^ Baseline characteristics, risk factors, angiographic findings, and types of PCI strategies were compared. The primary end-points including all-cause mortality, CV mortality, MI, and repeated PCI were also analyzed among the 4 groups. The beginning of follow-up was the date of index PCI procedure, and the duration of follow-up was from its beginning through December 2016 or the occurrence of any of the above primary end-points.

### 2.3 Statistical analysis

The analysis was used primarily to assess differences among groups. Analysis of variance was used to test continuous variables, and Chi-squared test or Fisher exact test was used to test categorical variables. Log-rank test and Kaplan–Meier curves were used to compare survival differences among groups. The Cox proportional hazards model was used to examine the effect of independent variables on hazards, and hazard ratio (HR) was used to describe the relative risk. *P* < .05 was considered significant. All analyses were performed using the statistical package SPSS for Windows, Version 23.0 (IBM Corp, Armonk, NY).

## 3 Results

During the 8-year study period, a total of 1676 patients with stable CAD who underwent a successful PCI procedure were enrolled. Among them, 445 patients in the control group had neither DM nor hypercholesterolemia, 376 patients had DM alone, 536 patients had hypercholesterolemia alone, and the remaining 319 patients had both DM and hypercholesterolemia. Patients with DM alone had the shortest follow-up time (control group, 41.8 ± 23.1 months; hypercholesterolemia alone, 51.2 ± 27.5 months; DM alone, 41.1 ± 23.8 months; and both hypercholesterolemia and diabetes, 45.8 ± 27.8 months; *P* < .01).

General characteristics of the study groups are listed in Table 1. There was no difference in age or body habitus among the 4 groups. As for the hemodynamic parameters, patients with both DM and hypercholesterolemia had the highest central pulse pressure (CPP) compared with patients in the other groups (*P* < .01). As for baseline biochemistry, patients with DM alone had the lowest cholesterol levels, the lowest LDL levels, and the worst renal function (all *P* < .01). Demographic data for the study population are shown on Table 2. Female and HT were more likely in patients with DM and hypercholesterolemia (both *P* < .01). However, patients with DM alone had the highest prevalence of CKD and were the least likely to be current smokers (both *P* < .01). In terms of medication after PCI, we found that patients with DM alone had the lowest rate of aspirin use (*P* < .01), whereas patients with both hypercholesterolemia and DM had the highest rate of diuretics use (*P* < .01). Besides the control group, patients with DM alone had the lowest usage of statins after PCI (*P* < .01).

**Table 1:**
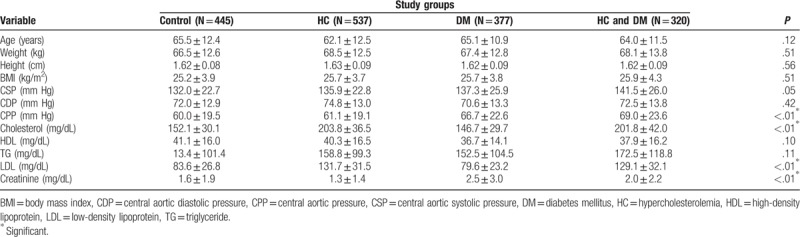
General characteristics of the study population among groups.

**Table 2:**
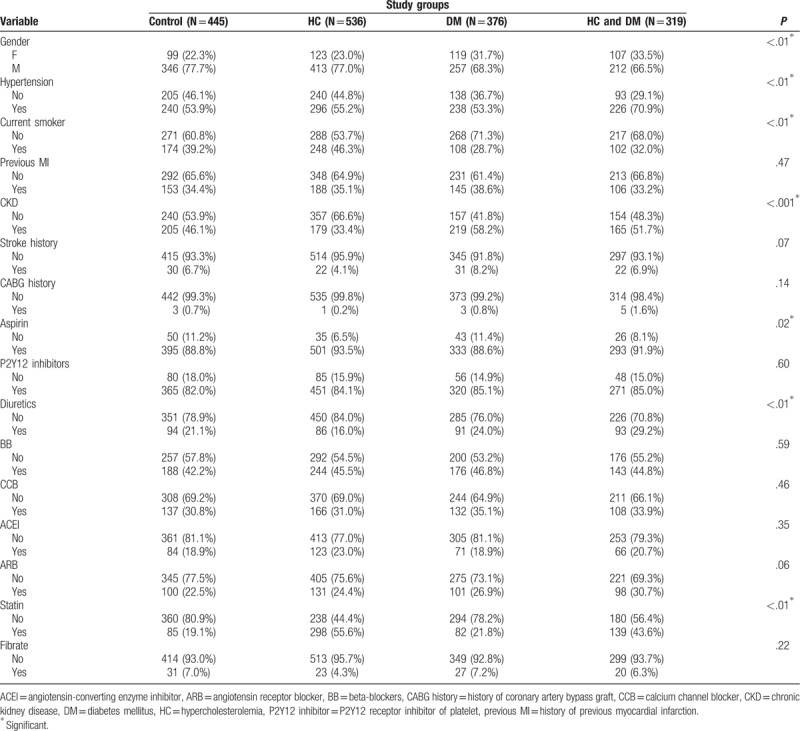
Demographics of the study population, and medications prescribed after index percutaneous coronary intervention among groups.

**Figure 1:**
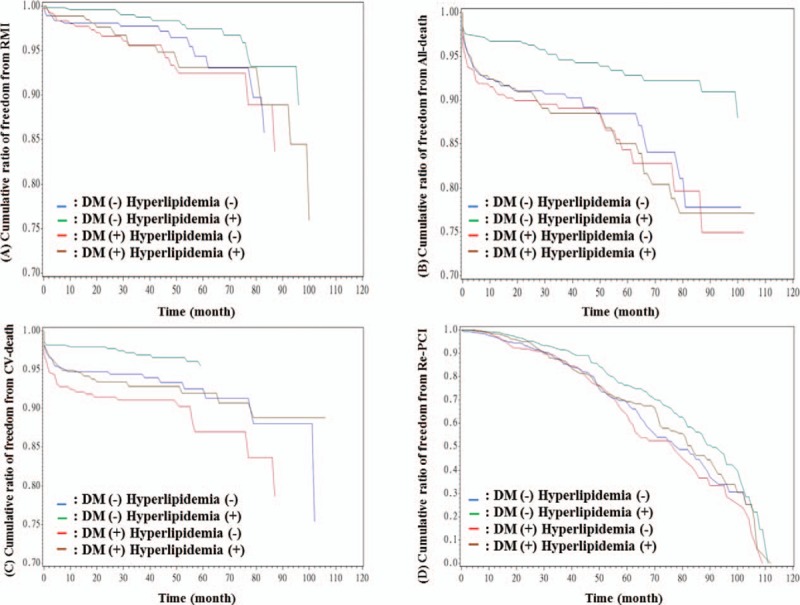
(A) Cumulative ratio of freedom from RMI between 4 groups (*P* = .029). (B) Cumulative ratio of freedom from all-death between 4 groups (*P* < .001). (C) Cumulative ratio of freedom from CV-death between 4 groups (*P* < .001). (D) Cumulative ratio of freedom from re-PCI between 4 groups (*P* = .003). CV = cardiovascular, DM = diabetes mellitus, PCI = percutaneous coronary intervention, RMI = recurrence of myocardial infarction.

Results of the angiographic findings and clinical outcomes are shown in Table 3. From the angiographic findings, patients with DM alone had the highest prevalence of double-vessel disease, while triple vessel disease was found most frequently in patients with both hypercholesterolemia and DM (*P* < .01). Patients with both hypercholesterolemia and DM also had the highest SYNTAX score (*P* < .01). As for adverse outcomes, patients with DM had the highest rate of CV mortality (*P* < .01) whereas patients with both hypercholesterolemia and DM had the highest rate of all-cause mortality (*P* < .01); however, the 4 groups had no significant difference in MI or repeated PCI rates. Figure 1 reveals the cumulated rate of freedom from MI, CV death, all-cause death, and repeated PCI among the 4 groups. Based on the Kaplan–Meier survival curve, freedom from all-cause mortality and CV mortality were lowest in the group with both hypercholesterolemia and DM, and the DM alone group, respectively (both *P* < .001). Figure 1 also showed the cumulative rate of freedom from MI, CV-death, all-cause death, and repeated PCI among 4 groups. Freedom from all-cause death, CV death was lowest in the DM alone group (both *P* < .001).

**Table 3:**
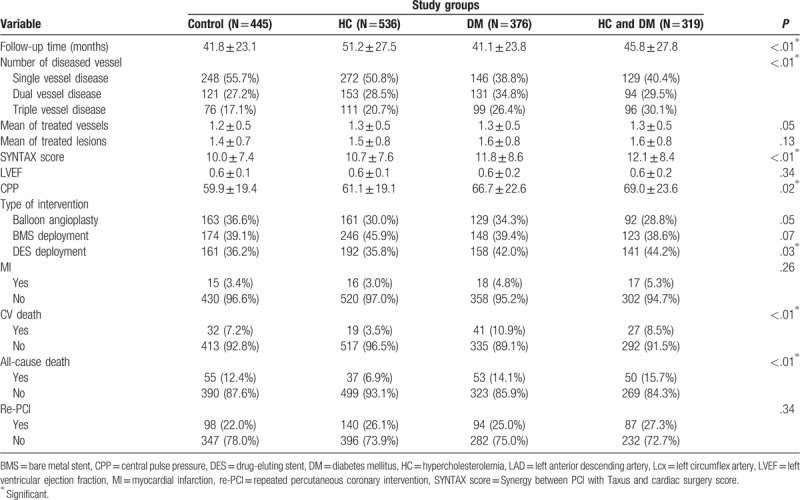
Demography of angiographic findings and outcome among groups.

An outcome analysis from the Cox proportion hazard model for MI, all-cause death, CV death, and repeated PCI is listed in Table 4. Compared with the control group, patients with both hypercholesterolemia and DM carried the highest risk for all-cause mortality (HR:1.70, 95% confidence interval:1.07–2.68, *P* < .05). In addition, we found that previous MI and SYNTAX scores were both predictors of MI (HR: 2.80 and 1.03, respectively). Age, previous MI, presence of CKD, and SYNTAX score were all predictors of all-cause mortality (HR: 1.04, 2.92, 2.56, and 1.03, respectively). Use of beta-blockers (BB), angiotensin-converting enzyme inhibitors (ACEI), and statins could reduce the risk (HR: 0.60, 0.54, 0.44, respectively). Age, previous MI, presence of CKD, SYNTAX score, and usage of P2Y12 receptor inhibitors of platelet (P2Y12 inhibitors) were the predictors of CV mortality (HR: 1.03, 3.75, 1.82, 1.03, and 2.23, respectively). DES deployment, usage of BB, ACEI, and statins could reduce the risk of CV mortality (HR: 0.56, 0.58, 0.46, and 0.51, respectively). Finally, smoking, previous MI, and presence of CKD were associated with repeated PCI (HR: 1.56, 1.29, and 1.54, respectively), whereas usage of ACEI could reduce the risk of repeated PCI (HR: 0.66).

**Table 4:**
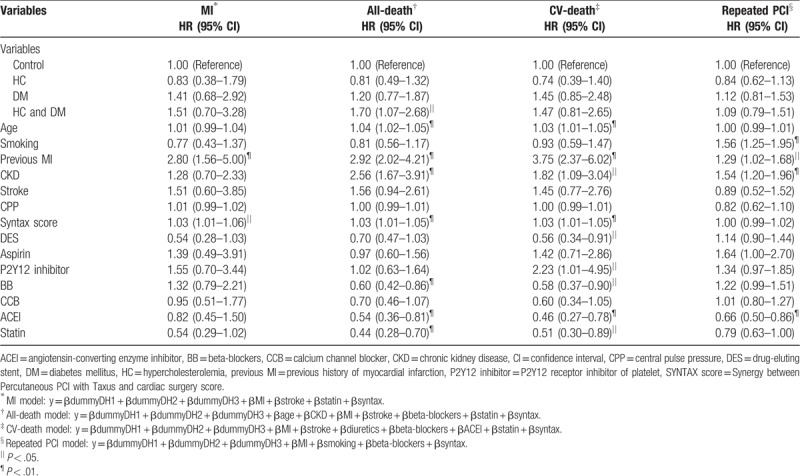
Significant predictors of outcome in Cox proportion hazard model for myocardial infarction, all-death, cardiovascular-death, and repeated percutaneous coronary intervention.

## 4 Discussion

Hypercholesterolemia and diabetes seemed to have an additive effect on all-cause mortality in patients with CAD after receiving PCI. However, whether comorbid with hypercholesterolemia or not, diabetes still had an adverse impact on long-term outcomes. In addition, we found that previous MI and SYNTAX scores were predictors of MI attack. Age, previous MI, presence of CKD, and SYNTAX score were all predictors of all-cause mortality; BB, ACEI, and statin use could reduce the risk of all-cause mortality. Age, previous MI, presence of CKD, SYNTAX score, and usage of P2Y12 inhibitors were predictors of CV mortality; DES deployment, usage of BB, ACEI, and statins could reduce this risk. Smoking, previous MI, and the presence of CKD were associated with repeated PCI, but usage of ACEI could reduce the risk of repeated PCI.

In our study, there was no difference in age or body habitus among the 4 groups. Patients with hypercholesterolemia alone and patients with both hypercholesterolemia and DM had higher serum cholesterol and LDL levels than those in the other 2 groups. In addition, whether comorbid with hypercholesterolemia or not, DM patients had higher creatinine levels than those in the other 2 groups. Other studies have found DM associated with development of CKD in older adults.^[17]^ Secondly, compared with patients with hypercholesterolemia, patients with both DM and hypercholesterolemia had a higher CPP, suggesting that DM has an additive effect on the progression of arterial stiffness in patients with hypercholesterolemia. Previous studies have shown that elevated CPP had a negative impact on CV outcomes in patients with HT and patients with CAD after undergoing a PCI procedure.^[18,19]^ The groups with DM included more females and CKD cases, therefore they used diuretics more frequently than the other groups; while the group with both hypercholesterolemia and DM included more HT cases, there was no difference as for anti-HT agents usage compared with the other 3 groups. Also, the group with DM alone had the lowest serum total cholesterol and LDL levels; it also used statins less frequently than the other 3 groups. Although statin use when LDL is less than 70 mg/dL has been found to improve CV outcomes in CAD patients after ACS,^[20]^ it is still unclear whether statin use may improve outcomes in DM patients with normal cholesterol and LDL levels.

In addition, patients with DM alone and those with both hypercholesterolemia and DM had higher rates of multivessel disease and higher SYNTAX scores; they also had a higher rate of DES usage during PCI. Compared with patients with hypercholesterolemia alone, patients with both hypercholesterolemia and DM had a significantly higher risk of developing multivessel disease. However, when compared with patients with DM alone, patient with both hypercholesterolemia and DM did not have a significantly higher risk of developing multivessel disease (*P* = .003). However, when compared with patients with DM alone, patient with both hypercholesterolemia and DM did not have a significant risk of developing multivessel disease (*P* = .28). This result is in contrast to a previous study, which observed a significantly higher risk of developing multivessel disease in patients with both DM and CKD, as compared with patients with DM alone.^[21]^ DM seemed to increase the risk of developing multivessel disease in patients with hypercholesterolemia, but hypercholesterolemia seemed not to increase this risk in patients with DM. Moreover, the 4 groups did not differ in terms of treated number of vessels and lesions; thus, whether with hypercholesterolemia or not, the presence of multivessel disease and high SYNTAX score in DM patients may affect long-term mortality. Patients with DM alone had the highest rate of CV mortality, while patients with both hypercholesterolemia and DM had the highest rate of all-cause mortality. Although statin use was not rare in patients with both hypercholesterolemia and DM, they still had a higher prevalence of multivessel disease, along with a higher SYNTAX score, and a higher prevalence of CKD, which may cancel out the benefit of statin therapy. In other studies, the usage of statins in patients with ACS could improve long-term outcomes,^[22–24]^ but the long-term benefit in stable CAD patients undergoing PCI remains to be clarified. On the other hand, compared to patients with hypercholesterolemia alone, patients with DM alone had an increased rate of all-cause mortality and CV mortality. We found patients with DM alone had a higher incidence of multivessel disease and CKD (both *P* < .001), along with higher SYNTAX scores compared with patients with hypercholesterolemia alone (*P* = .042). As has been stated, endothelial dysfunction and inflammation lead to the progression of atherosclerosis in patients with DM as well as patients with hypercholesterolemia^[25,26]^; whether the 2 conditions have an additive effect on endothelial dysfunction and aggravation of inflammation remains to be determined.

Nevertheless, there were several limitations in this study. First, the intensity of medication, such as tight lipid control and tight blood glucose control, were not surveyed in this study, which may affect long-term outcomes. Second, data entry bias cannot be ruled out; since functional or anatomic evaluations of the atherosclerotic lesions such as fraction flow reserve or intravascular ultrasound were not routinely used in this study, index PCI enrollment may have been affected. Third, since the number of patients with both hypercholesterolemia and DM was fewer than that of the other groups, the study power may have been affected. Fourth, since the future event rate in patients with stable CAD undergoing PCI is lower than that of patients with ACS, inadequate follow-up time cannot be excluded in this study.

In conclusion, whether comorbid with hypercholesterolemia or not, diabetes has an adverse impact on long-term outcomes in patients with stable CAD receiving PCI. DM seemed to be a more hazardous outcome predictor than hypercholesterolemia. Hypercholesterolemia and diabetes seemed to have an additive effect on all-cause mortality in patients after receiving PCI.

## Author contributions

**Conceptualization:** Mao-Jen Lin, Tzu-Yao Chuang, Han-Ping Wu.

**Formal analysis:** Chun-Yu Chen.

**Funding acquisition:** Mao-Jen Lin.

**Investigation:** Mao-Jen Lin.

**Methodology:** Mao-Jen Lin, Han-Ping Wu.

**Project administration:** Han-Ping Wu.

**Resources:** Mao-Jen Lin.

**Software:** Yu-Ching Chang.

**Supervision:** Han-Ping Wu.

**Validation:** Chia-Chen Huang, Tzu-Yao Chuang.

**Visualization:** Han-Ping Wu.

**Writing – original draft:** Mao-Jen Lin.

**Writing – review & editing:** Mao-Jen Lin, Tzu-Yao Chuang.
